# Anti-Diabetic Effects of Acankoreagenin from the Leaves of *Acanthopanax Gracilistylus* Herb in RIN-m5F Cells via Suppression of NF-κB Activation

**DOI:** 10.3390/molecules23040958

**Published:** 2018-04-19

**Authors:** Man-Xia Lu, Yang Yang, Qin-Peng Zou, Jiao Luo, Bin-Bei Zhang, Xiang-Qian Liu, Eun-Hee Hwang

**Affiliations:** 1 School of Pharmacy, Hunan University of Chinese Medicine, Changsha 410208, China; sweetlumanxia@163.com (M.-X.L.); LJhnzyy@163.com (J.L.); surblue77@163.com (B.-B.Z.); 2 School of Life Sciences, Datong University, Datong 037009, China; dwxlll215@163.com; 3 Changsha Broad-Ocean Bio-Science and Technique Co., Ltd., Changsha 410205, China; zouqinpeng@163.com; 4 Department of Food and Nutrition, Wonkwang University, Iksan 54538, Korea

**Keywords:** *Acanthopanax gracilistylus*, acankoreagenin, anti-diabetic effects, α-glucosidase, α-amylase, PTP1B, RIN-m5F cells

## Abstract

Diabetes mellitus is a chronic degenerative disease that causes long-term complications and represents a serious public health problem. In this manuscript, acankoreagenin isolated from the leaves of *Acanthopanax gracilistylus* (LAG) is thought to possess excellent anti-diabetic properties. In vitro, anti-diabetic activities were assessed based on the inhibitory activities with α-glucosidase (IC_50_ 13.01 μM), α-amylase (IC_50_ 30.81 μM), and PTP1B (IC_50_ 16.39 μM). Acankoreagenin showed better anti-diabetic effects. Then, an investigation was performed to analyze the insulin secretion effects of the insulin-secreting cell line in RIN-m5F cells. It was found that acankoreagenin could increase the insulin release in RIN-m5F cells. It was also found that acankoreagenin reduced NO production, activity of caspase-3, and the reactive oxygen species levels in the cells injured by processing of cytokines. In western blotting, inactivation of NF-κB signaling was confirmed. Acankoreagenin (20 μM) showed a higher I-κBα expression and lower NF-κB expression than the control group and showed a better expression than the positive control L-NAME (1 mM) (*p* < 0.05). This study demonstrates the anti-diabetic effects of acankoreagenin in vitro and suggests acankoreagenin might offer therapeutic potential for treating diabetes mellitus.

## 1. Introduction

Diabetes mellitus (DM) is considered one of the five leading causes of death in the world. The increased prevalence of diabetes has topped the list of global epidemic health concerns worldwide. Globally, an estimated 422 million adults were living with diabetes in 2014 compared to 108 million in 1980. The global prevalence (age-standardized) of diabetes has nearly doubled since 1980 rising from 4.7% to 8.5% in the adult population. There are two types of diabetes mellitus: Type 1 is the lack of insulin due to a destructive process in pancreatic β-cells and Type 2 is a steady decline in the use of glucose due to resistance of the tissues to insulin [1].

Type 1 diabetes (T1DM) is an autoimmune disease that is induced by selective destruction of insulin producing β-cells of the Langerhans islets [2,3]. Insulin-secreting β-cells failure is a common feature of diabetes, which leads to relative or absolute insulin deficiency. The inflammatory mediators have been concerned as having a crucial role in the prolonged suppression of β-cells apoptosis, β-cells function, and progressive β-cells loss [4]. Evidence is growing that interleukin (IL)-1β, tumor necrosis factor α (TNF-α), and interferon γ (IFN-γ) are candidate cytokines that participate in promoting β-cells death with some combined effects [5,6]. The mechanism underlying inflammation and β-cells death observed in T1DM conditions appears to involve the activation of NF-κB, which is activated by inflammatory responses during viral and bacterial infections. It is involved in expression of iNOS genes [7,8]. NF-κB activity is increased in cytokine-treated β-cells and IL-1β exerts its main effects through the transcriptional NF-κB pathway [9,10]. NF-κB is initially located in the cytoplasm as an inactive form with I-Bκα, which is an inhibitory factor of NF-κB [11,12]. NF-κB then translocates to the nucleus where it interacts with specific DNA recognition sites to mediate gene transcription [13]. Rodent β-cells synthesize two different insulins that are encoded by two nonallelic genes with more than 90% homology. The primary translation products, which are called preproinsulin I and II, differ by three amino acids in the pre-region, two amino acids in the C peptides, and two amino acids in the B chain. The conversion products insulin I and II are usually stored in unequal amounts [14]. IRS-1 was the first substrate identified and represents the prototype of the IRS family proteins. IRS-I is responsible for glucose uptake (skeletal muscle and adipose tissue) and IRS-II is responsible for glucose production (liver) and insulin production (pancreatic β-cell). In addition, IRS-I appears to have its major role in skeletal muscle where IRS-II regulates hepatic insulin action as well as pancreatic β-cell development and survival. By contrast, IRS-III genes appear to play a redundant role in the IRS signaling system [15]. Blockage of any of the NF-κB-iNOS-NO pathways would be useful in preventing the death or dysfunction of β-cells exposed to the cytokine mixture (IL-1β and IFN-γ).

Approximately 90% of people with diabetes around the world have Type 2 diabetes (T2DM), which is largely the result of excess body weight and physical inactivity. It has been demonstrated that, in this type of diabetes, the blood sugar rises abnormally right after a meal. Therefore, the control of the blood postprandial glucose level is an important factor in type 2 diabetes [16]. α-Amylase and α-glucosidase are the key enzymes that catalyze hydrolysis of α-glucosidic bonds in complex carbohydrates, like starch, to liberate absorbable glucose [17,18]. Protein tyrosine phosphatase 1B (PTP1B) interacts with the activated IR and with insulin receptor substrate (IRS) proteins. Afterward, it dephosphorylates tyrosine residues on the IR and IRS proteins [19,20,21]. Therefore, inhibitors of PTP1B are predicted to be useful for preventing and treating T2DM in vitro.

Around the world, many traditional plants have been found to successfully treat diabetes. *Acanthopanax gracilistylus* (AG) is widely distributed in China and the root bark, which has been listed in the Chinese pharmacopoeia, is used as medicine for treating rheumatism, paralysis, arthritis, sinew, bone pains, and as a tonic in traditional Chinese medicine [22,23]. In recent years, researchers found that the leaves of AG (LAG) contain diterpenoids, lignans, triterpenoids, polyacetylenes, phenylpropanoids, and flavonoids [24,25,26,27,28,29,30,31,32,33]. One previous pharmacological study on AG reported anti-tumor, anti-inflammatory, liver protective effects and suppressive effects on human lymphocytes [34,35,36]. Recently, the research of Zhang et al. indicated that acankoreagenin from LAG could significantly attenuate the release of high mobility group box chromosomal protein 1 and suggests this component as a candidate therapy for fulminant hepatitis [37]. Similarly plant-derived lupane-triterpenoids such as ursolic acid [38] have shown anti-inflammatory or antidiabetic effects. For acankoreagenin belonging to lupane-triterpenoids, we speculate that it might have anti-diabetic effects, but to the best of our knowledge, there is no relevant information reporting this.

Therefore, this study investigated acankoreageninon and its anti-diabetic enzyme activities with α-glucosidase, α-amylase, and PTP1B inhibitory activities. Then the insulin secretion effects of RIN-m5F cells were investigated.

## 2. Results

### 2.1. Abilities of the Compound Acankoreagenin from LAG to Inhibit α-Glucosidase, α-Amylase, and PTP1B

The anti-diabetes enzymatic activities of acankoreagenin were evaluated. As shown in Table 1, there was a higher *α*-glucosidase inhibitory activity with an IC_50_ value of 13.01 ± 0.38 μM, which had a better suppressed activity than the positive control known as acarbose [39]. It also showed the higher *α*-amylase inhibitory activity with an IC_50_ value of 30.81 ± 1.04 μM. On the PTP1B inhibitory activity, it showed an IC_50_ value of 16.39 ± 0.54 μM. It also illustrated higher suppressed activity than the positive control known as ursolic acid.

### 2.2. Cell Viability

**Figure 1:**
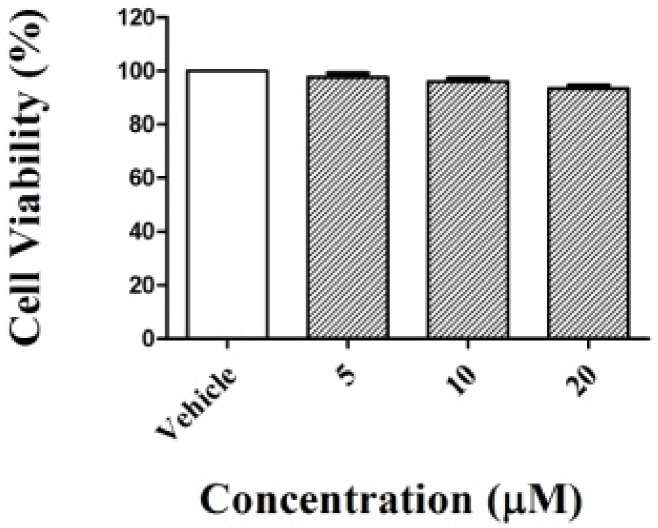
Viability of cells treated with acankoreagenin by the MTT assay. RIN-m5F cells were treated with various concentrations of acankoreagenin and the cytotoxicity level was determined by the MTT assay. Bars indicate SEM (*n* = 3).

The effect of the compound acankoreagenin from LAG on viability in RIN-m5F cells was presented in Figure 1. Cells were treated with acankoreagenin (5 μM, 10 μM, and 20 μM) for 24 h. All samples showed no cytotoxicity. Therefore, we used these concentrations in the following experiments.

### 2.3. Effects of Acankoreagenin on GSIS in RIN-m5F Cells

**Figure 2:**
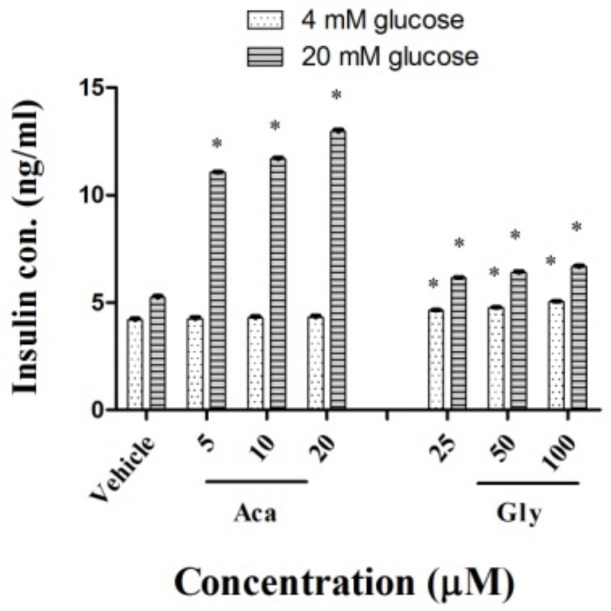
Effects of acankoreagenin on glucose stimulated insulin secretion. RIN-m5F cells were either cultured in basal (4 mM) or stimulated (20 mM) glucose concentrations in the presence of samples. * *p* < 0.05 versus vehicle-treated control. Bars indicate SEM (*n* = 3). Gly, glybunide; Aca, acankoreagenin.

The effects of acankoreagenin induced significantly (*p* < 0.05) in dose-dependent increments in insulin secretion of RIN-m5F cells under both basal (4 mM) and stimulated (20 mM) glucose concentrations are shown in Figure 2. The effect of it on the insulin release under a glucose challenge was significantly higher than that in the basal state. These results demonstrated that acankoreagenin increased insulin release in a dose dependent manner with 11.05 ± 0.12 ng/mL, 11.68 ± 0.11 ng/mL, and 12.92 ± 0.1 ng/mL at concentrations of 5 μM, 10 μM, and 20 μM, respectively, which were stronger than the positive control glybunide [40] at aconcentrations of 25 μM, 50 μM, and 100 μM. Therefore, it may have an anti-diabetic effect through β-cells secreting insulin.

### 2.4. Effects of Acankoreagenin on the Expression of Insulin Secretion-Related Gene in RIN-m5F Cells

**Figure 3:**
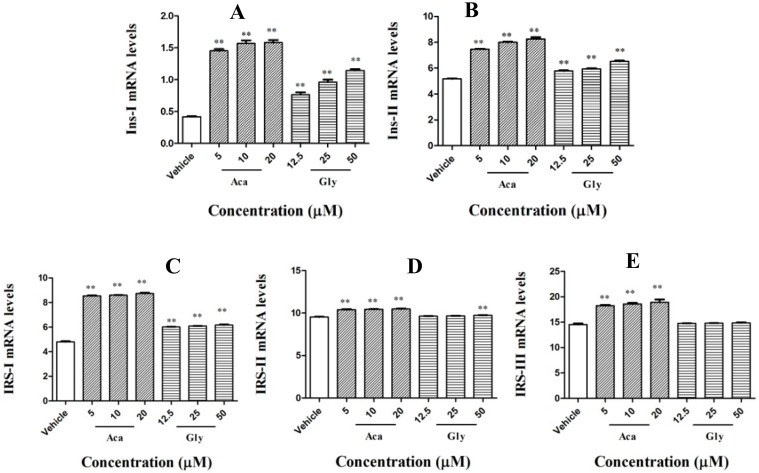
Effects of acankoreagenin on the expression of insulin secretion-related gene in RIN-m5F cells. Expression was determined after culturing the cells in six-well plate containing 20 mM glucose and then treated in the absence (vehicle) or various concentrations of acankoreagenin for 24 h. Expression levels were analyzed by real-time RT-PCR. β-actin mRNA was used as an internal control. ** *p* < 0.01 versus vehicle-treated cells. Bars indicate SEM (*n* = 3).Gly, glybunide; Aca, acankoreagenin. (**A**) Ins-I mRNA expression; (**B**) Ins-II mRNA expression; (**C**) IRS-I mRNA expression; (**D**), IRS-II mRNA expression; (**E**), IRS-III mRNA expression.

In marked contrast, the increment of acankoreagenin in Ins-I mRNA expression was significantly greater than vehicle-treated cells, which is seen in Figure 3A (*p* < 0.01).The increment of it in Ins-II mRNA expression was significantly greater than vehicle-treated cells, which was shown in Figure 3B (*p* < 0.01). The increment of it in IRS-I mRNA expression was significantly greater than vehicle-treated cells, which is shown in Figure 3C (*p* < 0.01). It also significantly increased IRS-II mRNA expression and IRS-III mRNA expression in a dose dependent manner. Therefore, it showed greater IRS-II mRNA expression and IRS-III mRNA expression than the positive control, which is shown in Figure 3D and Figure 3E (*p* < 0.01).

### 2.5. Effects of Acankoreagenin on the Cytokine-Induced NO Production in RIN-m5F Cells

**Figure 4:**
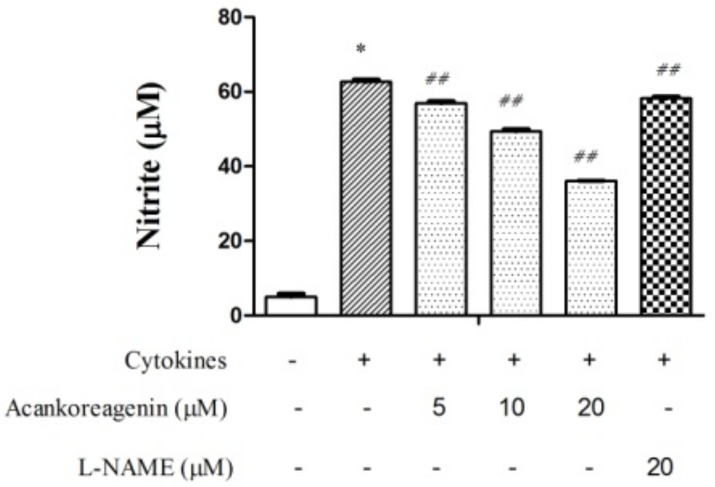
Effects of acankoreagenin on the cytokine-induced NO protection in the RIN-m5F cells.RIN-m5F cells were pretreated with the indicated concentrations of samples for 1 h and then exposed to IL-1β (10 ng/mL) and IFN-γ (100 ng/mL) for 24 h. Then the level of nitrite production was determined. * *p* < 0.05 versus vehicle-treated control; ^##^ *p* < 0.01 versus cytokine-treated group. Bars indicate SEM (*n* = 3).

As shown in Figure 4, NO production significantly increased in acankoreagenin when compared with the vehicle (*p* < 0.01). At a concentration of 20 μM, the level of NO production decreased to 36.1 ± 0.85%. These results demonstrated that acankoreagenin may decrease NO production in cytokines treated RIN-m5F cells in a concentration dependent manner. It showed a greater stimulated NO production effect than the positive control (20 μM). However, we did not restore viability back to the vehicle level.

### 2.6. Effects of Acankoreagenin on the Cytokine-Induced Cell Death in RIN-m5F Cells

**Figure 5:**
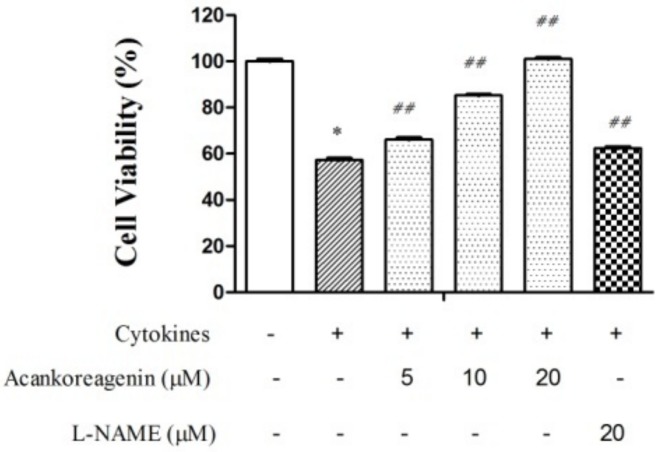
Effects of acankoreagenin on the cytokine-induced cell death in the RIN-m5F cells. The RIN-m5F cells were pretreated with the indicated concentrations of samples for 1 h and then IL-1β (10 ng/mL) and IFN-γ (100 ng/mL) were added. Cell viability was determined using the MTT assay. * *p* < 0.05 versus vehicle-treated control; ^##^ *p* < 0.01 versus cytokine-treated group. Bars indicate SEM (*n* = 3).

Compared to the vehicle, the cytokines treated RIN-m5F cells caused a significant reduction in cell viability to 57.61 ± 2.73% in Figure 5. It rescued cell viability to 106.34 ± 2.28% at a concentration of 20 μM (see Figure 5). Acankoreagenin is higher than the positive control, which rescued cell viability to 62.33 ± 1.85% at a concentration of 20 μM. These results demonstrated that acankoreagenin may partially preserve cell viability in cytokines treated RIN-m5F cells in a concentration dependent manner and it can restore viability back to the vehicle level.

### 2.7. Effects of Acankoreagenin on the Cytokine-Induced Caspase-3 Activity in RIN-m5F Cells

**Figure 6:**
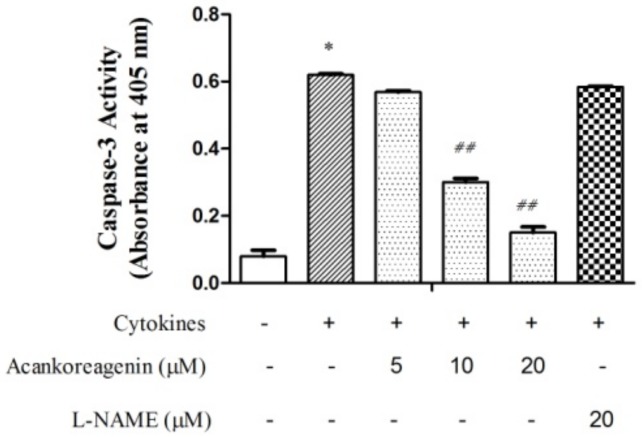
Effects of acankoreagenin on the cytokine-induced caspase-3 activity in the RIN-m5F cells. The RIN-m5F cells were pretreated with the indicated concentrations of samples for 1 h and then IL-1β (10 ng/mL) and IFN-γ (100 ng/mL) were added. Caspase-3 activity was based on spectrophotometric detection of the chromophore p-NA after cleavage from the labeled substrate DEVD-pNA. * *p* < 0.05 versus vehicle-treated control; ^##^ *p* < 0.01 versus cytokine-treated group. Bars indicate SEM (*n* = 3).

In order to investigate whether the observed rescue of the β-cell from apoptosis by acankoreagenin could be attributed to less activated caspase enzymes, we measured activity of an executioner, which is caspase-3. In RIN-m5F cells treated with cytokines and acankoreagenin at a concentration of 10 μM and 20 μM showed significantly lower caspase-3 activity when compared to the cytokines-treated cells, which is shown in Figure 6 (*p* < 0.01). These results demonstrated that acankoreagenin suppressed cytokine induced apoptosis by restricting the activity of caspase-3.

### 2.8. Effects of Acankoreagenin on the Cytokine-Induced ROS Levels in RIN-m5F Cells

**Figure 7:**
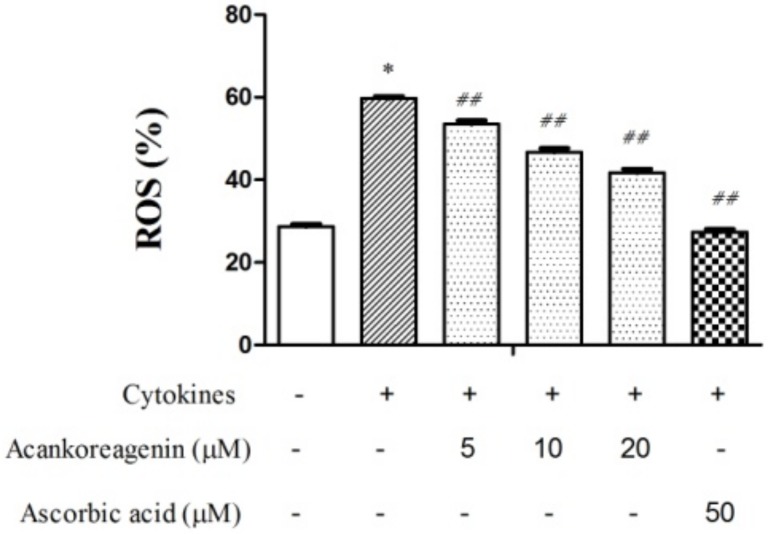
Effects of acankoreagenin on the cytokine-induced ROS levels in RIN-m5F cells. The RIN-m5F cells were pretreated with the indicated concentrations of samples for 1 h and then IL-1β (10 ng/mL) and IFN-γ (100 ng/mL) were added for 24 h. The intracellular ROS generated was detected by flow cytometry after 30-min incubation with an oxidation-sensitive probe DCFH-DA treatment. * *p* < 0.05 versus vehicle-treated control; ^#^ *p* < 0.05 and ^##^ *p* < 0.01 versus cytokine-treated group. Bars indicate SEM (*n* = 3).

The intracellular ROS were analyzed by using the oxidation-sensitive probe DCFH-DA in Figure 7. Flow cytometric analysis of RIN-m5F cells exposed to cytokines revealed a dramatic increase in ROS generation (56%). As shown in Figure 7, acankoreagenin showed a concentration dependent manner reduced the level of ROS to 53%, 46.67%, and 41.67% at a concentration of 5 μM, 10 μM, and 20 μM respectively. However, it showed lower than the positive control ascorbic acid (50 μM).

### 2.9. Effects of Acankoreagenin on the Cytokine-Induced Activation of NF-κB in RIN-m5F Cells

**Figure 8:**
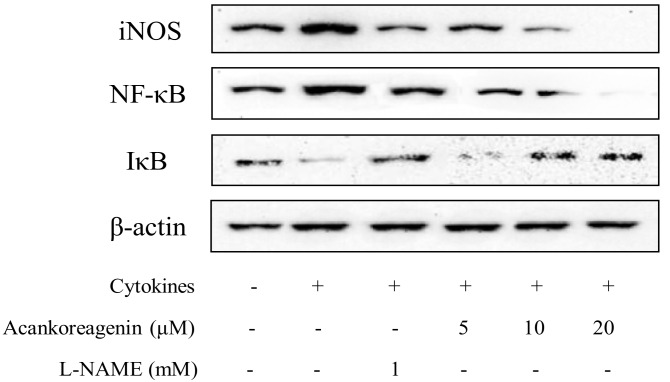
Effects of acankoreagenin on the cytokine-induced activation of NF-κB in RIN-m5F cells. RIN-m5F cells were pretreated with the indicated concentrations of samples for 1 h and then IL-1β (10 ng/mL) and IFN-γ (100 ng/mL) were added for 24 h. Then iNOS protein expression, I-κBα degradation, and activation of NF-κB p65 in RIN-m5F cells were determined by western blotting. β-actin was used as loading controls for cytosilic. Three independent experiments were done and all gave similar results.

The effects of acankoreagenin on the cytokines stimulated expression of iNOS protein expression and NF-κB p65 activation was examined by western blotting. The cytokine-treated RIN-m5F cells showed increased levels of iNOS and NF-κB protein expression as well as decreased levels of I-κBα protein expression in Figure 8. Acankoreagenin (20 μM) significantly reduced higher expression of protein levels of iNOS and NF-κB p65 and increased I-κBα degradation. These results demonstrated that acankoreagenin inhibited NF-κB activation by reducing NF-κB p65 activity and increasing I-κBα activity, which prevented iNOS expression.

## 3. Discussion

In the past few decades, the use of traditional Chinese medicine as a diabetes agent has gained much attention. In this study, we present for the first time the action mode of acankoreagenin from the LAG for protecting against diabetes development. Prior administration of LAG insulin secretion preserved functional β-cell mass. From the EA fraction of LAG, we isolated compound acankoreagenin. Afterward, anti-diabetic effects of acankoreagenin were tested with enzyme activities and investigated on the insulin secretion effect of RIN-m5F cells in vitro.

On the anti-diabeticenzyme activities with α-glucosidase, α-amylase, and PTP1B inhibitoryactivities, acankoreagenin showed the anti-diabetic effects.

In this study, the author used β-cell line RIN-m5F cells to determine the glucose-stimulated insulin secretion. Then, via qRT-PCR, the author determined acankoreagenin in insulin secretion gene expressions. Acankoreagenin significantly increased the expression of Ins I and II (see Figure 3A and Figure 3B). It also significantly increased the expression of IRS I and III, but showed no effect in the expression of IRS II (see Figure 3C–E). It showed a stronger insulin secretion than the positive control glybunide. The author observed that a 24 h exposure of cytokine combination (IL-1β (10 ng/mL) + IFN-γ (100 ng/mL)) severely impaired RIN-m5F β-cell and induced cell apoptosis. NF-κB regulates the expression of multiple pro-inflammatory genes such as iNOS [12]. Moreover, IL-1β in synergy with IFN-γ or alone was proposed for inducing NF-κB p65 translocation into the cell nucleus and for increasing the expression of iNOS mRNA and NO production [41]. NO is a short-lived and highly reactive radical. Besides its direct toxicity, NO reacts with superoxide to form peroxynitrite, which has much stronger oxidant activity and mediates β-cell destruction in type I diabetes [42]. Additionally, acankoreagenin showed better prevention of β cell damage effect than the positive control L-NAME (the iNOS inhibitor) [43].

The in vitro experiment does not consider the metabolism and pharmacokinetic factors and the effect of acankoreagenin is less clear in vivo. Moreover, in order to rule out the possibility that acankoreagenin produced these results due to interaction with cytokine directly, further in vivo experiments were conducted. Additional in vivo experiments are required to evaluate whether blocking β-cell NF-κB activation will indeed protect these cells against the immune response, which would lead to β-cell death in islets transplanted in animal models of type 1 diabetes.

## 4. Materials and Methods

### 4.1. Reagents

Ursolic acid (U6753, ≥90% pured by HPLC), glybunide (PHR1287-1G, Lot#LRAA9084 pharmaceutical secondary standard; traceable to USP, PhEur and BP) and L-NAME (N5751-250MG, Lot #BCBT1086, ≥98%) were purchased from Sigma-Aldrich (St. Louis, MO, USA).

### 4.2. Plant Materials and Isolation of Acankoreagenin from LAG

The leaves of *Acanthopanax gracilistylus* were collected at Changsha, Hunan province, China, in October 2015 and confirmed by Professor Xiang-Qian Liu at the Hunan University of Chinese Medicine who is one of the authors of this manuscript. A voucher specimen (no. 20151006) was deposited in the authorized laboratory.

**Figure 9:**
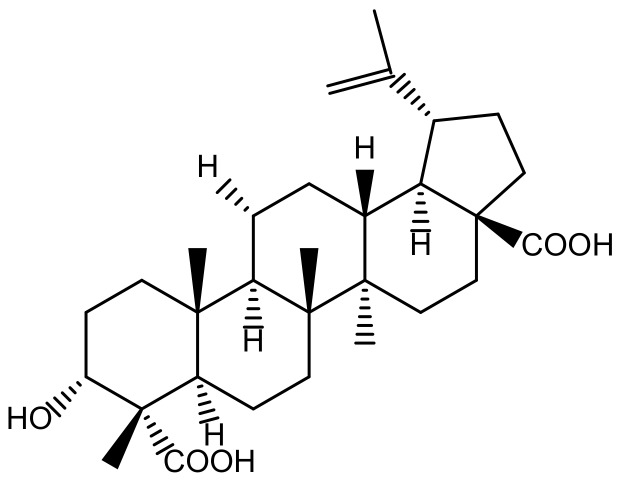
The chemical structure of acankoreagenin.

The following isolation method of acankoreagenin from LAG was described by Xiang-Qian Liu (one of the authors of this manuscript) [24]. The chemical structure of this compound was characterized on the basis of ^1^H-NMR (Bruker Co., Billerica, MA, USA), ^13^C-NMR (Bruker Co.), and X-ray (ELETTRA, Trieste, Italy) spectral analysis and comparisons with published spectral data [44]. The chemical structure of acankoreagenin was shown in Figure 9. The purity of the compounds was more than 98%, which was analyzed by HPLC (Aglient, Santa Clara, CA, USA).

### 4.3. α-Glucosidase, α-Amylase, and PTP1B Inhibition Assay

Theα-glucosidase inhibition assay was completed by using the method of Geng, S. et al. [45] with slight modification. Briefly, 20 μL of α-glucosidase (0.25 U) solution and 60 μL of samples were added and acarbose was used as a positive control. After thoroughly mixing, the samples were incubated at 37 °C for 18 min and then 50 μL of p-Nitrophenyl-α-d-glucopyranoside (pNPG) solution (5 mM) was added and then the mixture was further incubated at 37 °C for 25 min. The reaction was stopped by adding 120 μL of 0.1 M Na_2_CO_3_. The amount of liberated glucose was determined by the glucose oxidase method and absorbance was measured at 405 nm.

The α-amylase inhibition assay was done by using the method of Xu, J. et al. [17] with slight modification. Briefly, 125 μL with different concentrations of samples were incubated with 125 μL of α-amylase from porcine pancrease solution (3 U/mL) at 37 °C for 10 min. After pre-incubation, 125 μL of 2% starch solution was added into the tube and further incubated for 30 min. The reaction was stopped by adding 250 μL of 48 mM dinitrosalicylic acid reagent and immediately tubes were incubated for 15 min in a boiling water bath. Once cooled to room temperature, the mixture was diluted with 1.5 mL of distilled water and the absorbance was measured at 540 nm. Acarbose was used as a positive control.

The PTP1B inhibition assay was done by using the method of Uddin, Z. et al. [20] with slight modification. A PTP1B kit (human, recombinant) was purchased from BIOMOL International LP (Plymouth, PA, USA). The PTP1B colorimetric assay kit is designed to measure PTP1B activity for screening and profiling applications in a homogeneous assay with no time-consuming washing steps. To each of the 96 wells in a micro-titer plate (final volume: 100 μL) 10 μL substrate and PTP1B (2.5 ng/μL) was added with a buffer containing 50 mM citrate (pH 6.0), 0.1 M NaCl, 1 mM ethylenediaminetetraacetic acid, and 2 μL of 100 mM dithiothreitol with or without test samples. Then the samples were incubated for 1 h at room temperature and the absorbance was measured at 540 nm. Ursolic acid was used as a positive control.

All assays were done in triplicate. The inhibitory effect was calculated by the follow equation:

Inhibition (%) = (1 − A/B) × 100

A: Sample − Blank

B: Control − Blank

### 4.4. Cell Cultures

The insulin-secreting β-cell line RIN-m5F cells were obtained from the American Type Culture Collection (Rockville, MD, USA). The cells were grown in RPMI 1640 medium containing 10% of fetal bovine serum (Gibco-BRL, Grand Island, NY, USA) and 1% of antibiotic-antimycotic (Gibco-BRL). Cells were cultured at 37 °C and 5% CO_2_ in a humidified atmosphere.

### 4.5. MTT Assay for Cell Viability

The viability of RIN-m5F cells was determined by analyzing the reduction of 3-(4,5-dimethylthiazol-2-yl)-2,5-diphenyltetrazolium bromide (MTT) to formazan. Cells were cultured in the 48 well plates (Falcon, Franklin, NJ, USA) at a density of 2 × 10^5^ per well. After the designated treatment, 300 μL of MTT solution (0.5 mg/mL) was added. After 4 h, blue formazan crystals were resolved with 200 μL of dimethyl sulfoxide (DMSO). Absorbance was measured at 570 nm.

### 4.6. Glucose-Stimulated Insulin Secretion Assay (GSIS)

The amount of insulin secreted by RIN-m5F cells was determined by the method of Ding, Y. et al. [5] with slight modifications. RIN-m5F cells were seeded at a concentration of 2 × 10^5^ per well in the 48 well plates and allowed to attach overnight prior to acute tests. Then wells were washed three times with Krebs-Ringer bicarbonate buffer (KRB; 115 mM NaCl, 5 mM KCl; 2.5 mM CaCl_2_, 24 mM NaHCO_3_, 25 mM HEPES, 1 g/L BSA; pH 7.4) and pre-incubated for 1 h at 37 °C. In addition, cells were then incubated for 1 h in 1 mL KRB with 4 mM or 20 mM glucose and samples. After 1 h, supernatants were removed from each well and centrifuged (2000 rpm for 5 min, at 4 °C). Then the insulin concentration was determined with rat insulin ELISA kit (ALPCO Co, Salem, NH, USA).

### 4.7. qRT-PCR Analysis

According to the manufacturer’s instructions, total RNA was isolated by using the trizol reagent (Gibco). Then first-strand complementary DNA (cDNA) was generated by a random hexamer primer with a cDNA synthesis kit (Thermo Scientific, Hudson, NH, USA). The primer pairs were synthesized according to the method described previously [4,10]. The primers were purchased from Bioneer (Daejeon, Republic of Korea) and β-actin was used as an internal control. Reactions were carried out in 384-well plates with the ABI-Prism 7000 Sequence Detection System (Applied Biosystems) with Absolute QPCR SYBR Green Mixes (Applied Bio systems Inc., Foster, CA, USA). The thermal profile for the qRT-PCR was 95 °C for 10 min, followed by 40 cycles at 95 °C for 15 min and for 60 min at 60 °C. The primers included are listed in Table 2.

### 4.8. Cytokine Treatment

RIN-m5F cells (from six to 12 passages) were seeded at a concentration of 2 × 10^5^ per well in the 48 well plates. To induce RIN-m5F cell death, combined cytokines (recombinant human IL-1β 10 ng/mL and rat IFN-γ 100 ng/mL, R&D Systems, McKinley, MN, USA) were added and then treated with samples at different concentrations for 24 h [8].

### 4.9. Nitrite Determination and Prevention of Cytokine-Induced Cell Death

The level of NO was determined by assaying the concentration of nitrite in the whole cells extracts and the cell culture medium. The culture supernatant was removed and 100 μL portions were mixed with a 100 μL of griess reagent for 10 min at room temperature in the dark. Sodium nitrite was used to generate a standard curve. The optical density value of the samples at 520 nm was measured. Results were indicated as the NO to protein ratio and were expressed as micromoles of NO per gram of protein. Prevention of cytokine-induced cell death was measured by MTT assay. After the designated treatment, 300 μL of MTT solution (0.5 mg/mL) was added. After 4 h, blue formazan crystals were resolved with 200 μL of DMSO. Absorbance was measured at 570 nm.

### 4.10. Caspase-3 Assay

To assess caspase-3 protease activity in cell lysates of RIN-m5F cells (2 × 10^5^ per well in the six well plates) after 24 h of treatment, we used the Caspase-3 Colorimetric Assay Kit (Abcam Biotech, Cambridge, MA, USA). The assay is based on spectrophotometric detection of the chromophore p-nitroaniline (p-NA) after cleavage from the labeled substrate DEVD-pNA. The p-NA light emission can be quantified using a micro-titer plate reader at 405 nm.

### 4.11. Assay of Intracellular Reactive Oxygen Species (ROS) Levels

To analyze the intracellular generation of ROS, cells were detached by trypsinization. After cells were treated with the sample for 24 h, the cells were treated at 37 °C for 20 min with the 10 μM oxidation-sensitive probe 2′,7′-dichlorodihydrofluorescein diacetate (DCFH-DA) by using a FACScantm flow cytometer (BD Biosciences, San Jose, CA, USA). After washing with phosphate buffered solution (PBS), 20,000 cells were detected with flow cytometry and then Flow-Jo 7.6 software (Cell bio. London, UK) was utilized to examine the level of intracellular ROS. Ascorbic acid was used as a positive control.

### 4.12. Western Blotting Analysis

Cell lysis was performed in 30 μL ice-cold lysis buffer (iNtRON Biotech, Scottsdale, AZ, USA) to the cell cultures. The resultant protein extracts were quantified and proteins (25 μg) were resolved by 10% sodium dodecyl sulfate polyacrylamide gel electrophoresis (SDS-PAGE) and then transferred to polyvinylidene difluoride (PVDF) membranes for 2 h. Membranes were blocked in 5% skim milk and probed with primary antibodies (1 μg/mL) against inhibitory κB (I-κBα), iNOS, NF-κB, and β-actin (Cell Signal Technology, Beverly, MA, USA) were incubated overnight at 4 °C. After five times, washing in Tris-buffered saline containing 0.1% Tween 20 (TBST), the membranes were incubated with anti-rabbit IgG antibodies for 2 h at room temperature. After washing in TBST five times, reactive bands were visualized using enhanced chemiluminescence (ECL) reagent. Protein expression was exposed by analyzing the signals captured on the PVDF membranes using a Fluor Cheme E image analysis (Cell bio.).

### 4.13. Statistical Analysis

All experiments were performed in triplicate. Dates were analyzed using the SPSS (Statistical Package for the Social Science, Ver. 18.0) program. The data are expressed as the mean ± SEM values. The differences between the means of the experimental and control groups were performed using the Student’s *t*-test. Additionally, comparisons between multiple groups were made by ANOVA and Duncan’s tests. Differences with a *p* value < 0.05 were considered statistically significant.

## 5. Conclusions

As a result, acankoreagenin from LAG showed the anti-diabetes enzymatic activities. It showed a better α-glucosidase, α-amylase, and PTP1B inhibitory activities (Table 1) than the positive control

Then, investigation was performed on the insulin secretion effects of insulin-secreting cell line RIN-m5F cells in vitro. This study demonstrated that acankoreagenin can increase the insulin release and showed a β-cell protective effect. Specifically, it protected the pancreas β-cell from cytokine-induced (IL-1β and IFN-γ) injury and inhibited NO production in RIN-m5F cells through suppression of iNOS in vitro. This pancreas β-cell protective effect may be mediated, at least in part, through the I-κBα signaling pathway and inactivation of NF-κB. These data indicated that acankoreagenin showed a beneficial effect when used to prevent the progress of diabetes.

Further investigations of the anti-diabetic effects of acankoreagenin are needed to be determined in diabetic rats in vivo [46].

## Figures and Tables

**Table 1:** Effects of acankoreagenin on α-glucosidase, α-amylase, and PTP1B inhibitory activities.

Compound	α-Glucosidase IC_50_ (μM)	α-Amylase IC_50_ (μM)	PTP1B IC_50_ (μM)
Acankoreagenin	13.01 ± 0.38 *	30.81 ± 1.04 *	16.39 ± 0.54 *
Acarbose ^1^	661.73 ± 0.48	854.43 ± 0.81	-
Ursolic acid ^2^	-	-	31.11 ± 0.47

Data of the 50% inhibition concentration (μM) were calculated from the inhibition curve and expressed as the mean ± SEM (*n* = 3). ^1,2^ Used as positive controls in each assay. * *p* < 0.05 when compared with the positive controls in each assay.

**Table 2:** Sequences of primer set.

Primer	Forward/Reverse
β-actin	TCTGAACCCTAAGGCCAACCGTG
	ATGGCATGAGGGAGCGCGTA
Insulin I (INS I)	CAAACAGCACCTTTGTGGTCCTCAC
	CACAATGCCACGCTTCTGCC
Insulin II (INS II)	CAGCACCTTTGTGGTTCTCACTTGG
	ATCCACGATGCCGCGCTTCT
Insulin receptor substrate I (IRS-I)	AGAACGAGAAGAAGTGGCGGCAC
	TGCAGCTGCAGAAGAGCCTG
Insulin receptor substrate II (IRS-II)	AGCGAGAAGAAGTGGAAGAGCAAGG
	TGACCAAGTCGGTGAGTGCG
Insulin receptor substrate III (IRS-III)	CCATCTGAGGAAGCAGAAGTCCCA
	TGACGATCAGGTGGCGCTGA
